# Association between Mobile Phone Addiction Index and Sugar-Sweetened Food Intake in Medical College Students Stratified by Sex from Shanghai, China

**DOI:** 10.3390/nu13072256

**Published:** 2021-06-30

**Authors:** Shaojie Liu, Weiqiang Zhou, Jiangqi Wang, Bo Chen, Gengsheng He, Yingnan Jia

**Affiliations:** 1 School of Public Health, Key Lab of Public Health Safety of the Ministry of Education, Fudan University, Shanghai 200032, China; liushaojie@fudan.edu.cn (S.L.); 18211020091@fudan.edu.cn (J.W.); chenb@fudan.edu.cn (B.C.); gshe@shmu.edu.cn (G.H.); 2 Informatization Office, Fudan University, Shanghai 200032, China; wqzhou@fudan.edu.cn

**Keywords:** sugar-sweetened food, mobile phone addiction, medical college students

## Abstract

This study’s objective was to depict sugar-sweetened food (SSF) consumption in medical college students stratified by sex from Shanghai, China, and to explore the association between the Mobile Phone Addiction Index (MPAI) and SSF intake. The data were obtained from 1121 medical college students from the Fudan University, Shanghai, China, who took an online questionnaire investigation in December 2020. Data included demographics, the MPAI, the Nutrition Literacy Assessment Questionnaire (NLAQ), total and food expenditure per month, the International Physical Activity Questionnaire (IPAQ), and a food frequency questionnaire (carbonated beverages (CB), other sugar-based beverages (OSBB), sugar/chocolate). We evaluated the association between the MPAI and three types of SSF intake according to multivariate logistic regression analysis stratified by sex. The mean CB, OSBB, and sugar/chocolate intakes were, respectively, 65.66 mL/d, 74.20 mL/d, and 4.96 g/d in men and 30.42 mL/d, 71.48 mL/d, and 4.99 g/d in women. The MPAI was positively associated with SSF intake, regardless of sex. In men, the CB and OSBB odds ratios (ORs) were, respectively, 1.023 (95% CI: 1.004–1.042), 1.019 (95% CI: 1.001–1.038); and in women, the CB, OSBB, and sugar/chocolate ORs were, respectively, 1.026 (95% CI: 1.013–1.039), 1.020 (95% CI: 1.007–1.033), and 1.019 (95% CI: 1.006–1.032). Age, NLAQ, total expenditure, food expenditure, and total physical activity also were related to SSF intake. Age and the application capacity of the NLAQ were negatively associated with SSF intake, whereas comprehension capacity of the NLAQ, total and food expenditure, and total physical activity were positively associated with SSF intake. This study confirmed that SSF intake is widespread among medical college students from Shanghai, China, even if they have relatively high nutrition health literacy. From a public health perspective, it is necessary to reduce SSF intake in medical college students by decreasing the MPAI, controlling the total and food expenditure per month in high-consumption areas, and improving the application ability of the NLAQ. Further studies are needed to explore the MPAI and other potential factors that may influence SSF intake of college students by expanding the sample size of college students throughout China, and the causal association between them.

## 1. Introduction

Sugar-sweetened food (SSF) includes sugar-sweetened beverages (carbonated or noncarbonated beverages, soft drinks, sport drinks, fruit drinks, and milk tea) and foods (sugar, sweetened chocolate, and pastries), which have become the main daily source of added sugar around the world [1,2]. A growing body of literature documents the prevalence of SSF intake in children, adolescents, and adults, likely caused by the high content of added sugars, especially sucrose or fructose, which induce strong positive hedonic stimulation of the sweetness quality sensors [3]. The World Health Organization (WHO) recommends reducing the energy provided by free sugar in SSF to less than 5% of the daily energy intake for optimal health benefit [4]. Recent literature on this topic has indicated that SSF intake in children, adolescents, and adults exceeds the intake recommended by the WHO. In the United States, a survey showed that nearly 64% of young people, aged 2–19 years, drink sugar-sweetened beverages every day [5], which contributes to 8.4% of their total daily energy intake. Studies conducted by Brand-Miller and Aburto showed that sugar-sweetened beverage intake contributes to 5.5% and 8.3% of the total energy intake in children from Australia and Mexico, respectively [6,7]. An increasing trend in sugar-sweetened beverage intake, equivalent to 6.5% of the total daily energy intake, was also observed among adults in the United States [8,9]. Overconsumption of SSF has been shown to induce uncontrolled liver uptake of fructose, which leads to hepatic lipid accumulation and dyslipidemia, reduces insulin sensitivity, and elevates uric acid levels [10]. This dysregulation is related to a series of diseases, including weight gain and obesity [11], metabolic syndrome [12], type 2 diabetes [13], cardiovascular disease [14], fatty liver [15], and hyperuricemia [16].

Previous research established a critical role for screen time, including television viewing, in children’s SSF intake [17]. Prolonged television viewing time was positively associated with sugar intake [18]. With the advent of the Internet era, however, the usage of mobile phones has surpassed that of viewing television [19]. Few studies have suggested that the increase in mobile phone usage is linked to increased intake of SSF [20]. Yet, the amount of time spent using mobile phones alone does not fully reflect the physical, psychological, and behavioral characteristics of mobile phone users. The Mobile Phone Addiction Index (MPAI), developed by Leung [21] to characterize mobile phone addiction symptoms and provide a comprehensive assessment of addiction level, has been widely applied in various studies around the world [22,23]. This methodology, consisting of a 17-item MPAI scale, was used to categorize the four mobile phone addiction symptoms, including “inability to control craving”, “feeling anxious and lost”, “withdrawal/escape”, and “productivity loss”. To the best of our knowledge, until our present study, no research applied this methodology to explore the relationship between the MPAI and SSF intake.

In addition to MPAI, other potential factors also influence the intake of SSF from several studies: Zoellner et al. discovered health literacy-focused strategies were an effective way to decrease the sugar-sweetened beverage intake [24]; Chen et al. found an association between living expenditure and sugar-sweetened beverage intake [25]; Santaliestra-Pasias et al. found that physical activity was related to SSF intake [26]. These factors influencing SSF intake were also included in our research. Meanwhile, a lot of discussions have focused on SSF intake in children and adolescents. Compared with children or adolescents, college students, with their increasing independence and autonomy, face continuous challenges in choosing food [27]. Therefore, they are more likely to consume SSF than other age groups, although this problem has received scant attention in research.

Importantly, a U.S. study revealed that among young adults, sugar-sweetened beverage intake accounts for 9.3% of the daily energy intake in men and 8.2% of the daily energy intake in women [28,29]. This information suggested that different factors may influence SSF intake in men and women. Thus, this issue has been considered in the design of the present study, which was conducted in the form of a cross-sectional survey performed through an online questionnaire. The purpose of this study was to depict SSF consumption and explore the association between the MPAI and SSF intake in medical college students stratified by sex from Shanghai, China. Moreover, we also examined other potential factors influencing SSF intake, including age, physical activity, total expenditure, food expenditure, Body Mass Index (BMI), and nutrition health literacy.

## 2. Materials and Methods

### 2.1. Target Population

We employed random cluster sampling in our study. First, we randomly selected four colleges and departments from the Fenglin campus of Fudan University, Shanghai, China: the School of Public Health, the School of Basic Medicine, the Institute of Brain Science, and the Institute of Biomedicine. All students from these colleges and departments were our target research objects, with the exception of the first-year students, who studied and lived on other campuses of Fudan University. The study was conducted in accordance with the Declaration of Helsinki, and the protocol was approved by the Ethics Committee of Medical Research, School of Public Health, Fudan University (Project identification code: IRB#2019-01-0726S).

### 2.2. Questionnaire Design

The questionnaire was composed of different parts, including demographic information (age, sex, height, and weight), lifestyle behaviors (MPAI, Nutrition Literacy Assessment Questionnaire (NLAQ), total and food expenditure per month, International Physical Activity Questionnaire (IPAQ)), and a food frequency questionnaire (carbonated beverages (CB), other sugar-based beverages (OSBB), sugar/chocolate). Additionally, we designed two simple mathematical calculations for quality control of the questionnaire. The informed consent form was also included in the questionnaire.

### 2.3. Questionnaire Survey

We designed the survey as an online questionnaire, which was conducted anonymously to collect data of college students, in December 2020. The online questionnaire was operated by the “Wenjuanxing” platform (https://www.wjx.cn/app/survey.aspx, accessed on 12 December 2020), which provides functions equivalent to those of Amazon Mechanical Turk. The online questionnaire was distributed to the target research objects. The college students who agreed to take the survey and signed the informed consent voluntarily completed the online questionnaire and allowed the use of their data for our research purpose. We collected total of 1155 questionnaires; we excluded 22 questionnaires due to calculation errors in the quality control test and 12 questionnaires for incompleteness. Finally, we retained 1121 valid questionnaires for the study, which corresponded to an effective response rate of 98.06%.

### 2.4. Mesurements

#### 2.4.1. SSF

Three types of SSF intake were the outcome variables in this study. We collected information on SSF intake through the food frequency questionnaire. We selected the three most representative SSFs: CB, OSBB, and sugar/chocolate. The food frequency questionnaire for each type of SSF was designed as two questions, including “How often do you consume the SSF on average?” and “How many grams or milliliters of the SSF do you consume on average?” For each SSF, the food frequency questionnaire asked two questions to estimate the intake frequency and the amount per intake. The information on intake frequency (none, less than once a month, 1–3 times a month, 1–3 times a week, 4–6 times a week, once a day, 2 times a day, 3 times a day, and above) was converted into the number of intakes per day, corresponding to the following values: 0; 0.02; 0.07; 0.29; 0.71; 1; 2; and 3. Finally, the number of intakes per day and the amount per intake were used to calculate, in grams, the total daily intake of CB, OSBB, and sugar/chocolate. According to their median (10.5 mL/d, 35.0 mL/d, and 1.0 g/d, respectively), we categorized these food intakes into low or high intakes.

#### 2.4.2. MPAI

MPAI was the main research factor in this study. The MPAI was developed by Leung [21] to determine the symptoms of mobile phone addiction and provide a comprehensive assessment of mobile phone addiction level. This index has been widely applied in various studies around the world [22,23]. The 17-item MPAI scale (Table S1) was used to assess four mobile phone addiction symptoms, including “inability to control craving,” “feeling anxious and lost,” “withdrawal/escape,” and “productivity loss.” We converted answers to questions, such as “Do your friends and family complain about your use of the mobile phone?” or “Do you feel lost without your mobile phone?” into scores, with 1 = “not at all,” 2 = “rarely,” 3 = “occasionally,” 4 = “often,” and 5 = “always.”

#### 2.4.3. Covariate Assessment

##### NLAQ

The NLAQ, which can reach a maximum score of 65, was developed by Wang based on the Critical Nutrition Literacy of Guttersrud [30] and the Assessment Indicators System of Health Literacy of Zhang [31]. It consists of 13 items (Table S2), which were divided into three dimensions: acquisition capacity (score ty20), including questions such as: “Do I discuss food with others?” and “Do I often refer to the information in the media?”; comprehension capacity (score ty30), including questions such as: “Do I understand the concept of ‘balanced diet’?”; and application capacity (score yi15), including questions such as: “Am I willing to spend extra time or money on healthy meals?” We used the NLAQ, the reliability and validity of which has been previously demonstrated, to effectively evaluate the dietary health literacy of Chinese college students.

##### BMI

Height and body weight were filled out by the students themselves and were accurate to 0.1 cm and 0.1 kg in the questionnaire. The BMI (kg/m^2^) = weight (kg)/height^2^ (m^2^) was categorized into three levels based on the Working Group on Obesity in China [32]: underweight (<18.5 kg/m^2^), normal weight (18.5–23.9 kg/m^2^), and overweight and obese (≥24 kg/m^2^).

##### Total and Food Expenditure

Total and food expenditure per month were estimated by the students in the questionnaire. We took the average living expenses from their parents and subsidies from the school every month as the total expenditure per month and took what the college students spent on food and meals as the food expenditure per month. Total and food expenditure per month were translated into dichotomous variables, based on their median (2000 and 1000 CNY/month).

##### Physical Activity

The IPAQ of the questionnaire asked about three specific types of physical activity including walking; moderate-intensity activities, such as dancing, cycling, playing ping pong, and practicing tai chi; and vigorous-intensity activities, such as swimming or playing basketball and badminton. We assessed the IPAQ according to the average number of days during which they completed more than 10 min of vigorous-intensity activities, moderate-intensity activities, or walking in the past week, and recorded the duration of each type of physical activity. To help students better distinguish among the different types of physical activities, we made an example diagram for each type of physical activity. The IPAQ, which has widespread application in the international arena, can be used to measure the overall weekly physical activity of the public [33]. We used the following values to analyze each type of physical activity from the questionnaire: walking = 3.3 metabolism equivalents (MET); moderate-intensity activity = 4.0 MET; and vigorous-intensity activity = 8.0 MET. The formula of IPAQ was as follows: the total physical activity (MET/min/w) = the MET value of physical activity × the amount of time spent on physical activity per day (min/d) × the number of days of physical activity per week (d/w) [34]. We converted the continuous variables corresponding to the total physical activity into three categorical variables according to Kyu’s classification, which uses cutoff values of 600 and 4000 MET/min/w [35] as follows: low total physical activity (<600 MET/min/w), moderate total physical activity (600–4000 MET/min/w), and high total physical activity (≥4000 MET/min/w).

### 2.5. Statistical Analysis

We stratified the analysis by sex. Continuous variables were described as means ± standard deviation (SD). Categorical variables were described as frequencies (ratios). Chi-square test or t-test was employed in the equilibrium test between MPAI, age, NLAQ, BMI, total physical activity, CB, OSBB, sugar/chocolate, total expenditure, food expenditure, and sex. MPAI, age, NLAQ, BMI, total physical activity, total expenditure, and food expenditure were all used as independent variables, and three dichotomous variables (i.e., CB, OSBB, and sugar/chocolate) were used separately as dependent variables. We utilized a multivariate logistic regression model stratified by sex to examine the factors influencing SSF intake among medical college students by calculating the odds ratios (ORs) and corresponding 95% confidence intervals (95% CI). All statistical analyses were conducted in SAS software version 9.2 (SAS Institute Inc., Cary, NC, USA), and two-sided *p* values < 0.05 were considered statistically significant.

## 3. Results

Of the 1121 participants enrolled in the study, 368 (32.83%) participants were men, with an average age of 23.22 ± 3.32 years, and 753 (67.17%) were women, with an average age of 23.45 ± 3.39 years. The demographic characteristics of the participants stratified by sex are presented in Table 1. The participants of both sexes had equal distributions of age, NLAQ, consumption of OSBB, and food expenditure per month (*p* > 0.05). The mean intakes of CB, OSBB, and sugar/chocolate were, respectively, 65.66 mL/d, 74.20 mL/d, and 4.96 g/d among male participants, and 30.42 mL/d, 71.48 mL/d, and 4.99 g/d among female participants. The mean ± SD of MPAI score in men was 44.94 ± 12.08, and in women was 45.25 ± 11.87. There was no significant difference between the two groups (*p* = 0.682). The percentages of being overweight and obese (30.16%), high total physical activity (9.78%), and low total expenditure per month (47.83%) were significantly higher in male participants than in female participants. Conversely, female participants had considerably higher percentages of being underweight (22.05%) and had lower total physical activity (24.97%) but had higher total expenditure per month. Regarding their SSF intake, male participants consumed higher amounts of CB, whereas female participants had higher intake of sugar/chocolate (*p* < 0.05).

Table 2 presents the MPAI and other potential factors that may influence CB intake according to sex. After covariate adjustments, the analysis showed that the higher the MPAI, the higher the percentage of intake of CB, with an OR of 1.023 (95% CI: 1.004–1.042) for men and an OR of 1.026 (95% CI: 1.013–1.039) for women. In addition, in male participants, the percentage of participants with a high intake of CB increased with monthly food expenditure, resulting in an OR of 1.881 (95% CI: 1.078–3.281).

The MPAI and other potential factors that may influence the intake of OSBB are presented in Table 3. Four variables, including MPAI, age, total expenditure, and food expenditure, significantly influenced the intake of OSBB, in both male and female participants. We found a positive association between MPAI and the intake of OSBB, with an OR of 1.019 (95% CI: 1.001–1.038) in men and 1.020 (95% CI: 1.007–1.033) in women. Age was negatively associated with the consumption of OSBB, with an OR of 0.929 (95% CI: 0.865–0.997) in men and 0.948 (95% CI: 0.904–0.993) in women. The total and food expenditure per month were positively associated with the intake of OSBB (*p* < 0.05).

MPAI, age, NLAQ, and total physical activity were associated with the intake of sugar/chocolate (Table 4). In female participants, the MPAI increased the odds of a high intake of sugar/chocolate (OR = 1.019, 95% CI: 1.006–1.032). In addition, in women, there was a significant decrease in sugar/chocolate intake with the increase in age and application capacity of the NLAQ, with OR values of 0.948 (95% CI: 0.906–0.993) and 0.909 (95% CI: 0.846–0.977), respectively. In male participants, participants with higher comprehension capacity of the NLAQ and total physical activity independently had increased odds of a high intake of sugar/chocolate (OR = 1.082, 95% CI: 1.013–1.155) and (OR = 2.860, 95% CI: 1.047–7.812), respectively, whereas higher application capacity of the NLAQ decreased the odds of a high intake of sugar/chocolate (OR = 0.901, 95% CI: 0.813–0.998).

## 4. Discussion

To the best of our knowledge, this study is the first to characterize the current pattern of SSF consumption in medical college students according to sex and to explore the association between the MPAI and SSF intake in students from Shanghai, China. To date, most studies have addressed these issues in children or adolescents, but few have focused on college students [36,37]. Compared with children and adolescents, college students may consume higher SSF because of their characteristic independence and immaturity. Yet, little data related to college students have been available, and this topic has needed more attention. In summary, even among medical college students with relatively high nutrition health literacy, the intake of SSF is still not optimistic. The mean intakes of CB, OSBB, and sugar/chocolate were, respectively, 65.66 mL/d, 74.20 mL/d, and 4.96 g/d in male participants, and 30.42 mL/d, 71.48 mL/d, and 4.99 g/d in female participants. The male students consumed significantly higher amounts of CB, whereas the female students consumed higher amounts of sugar/chocolate. Analysis by multivariable logistic regression showed an obvious association between the MPAI and SSF intake.

With the popularity of Internet, mobile phones, as the most popular mobile Internet terminal, have become essential for human interaction, lifestyle, and access to media [38]. In China, the number of mobile phone users has reached 847 million and is still growing [38]. Although mobile phones bring convenience, some of their hazards are also becoming increasingly apparent. Soni et al. [39] demonstrated that excessive use of mobile phones can develop into serious sleep and behavior problems, and our research also confirmed this finding. The most important finding of this study was that the MPAI was positively associated with CB and OSBB, regardless of sex, and with sugar/chocolate intake in women. This finding is in keeping with and confirms the results obtained in another study [17]. The use of the MPAI allowed us to evaluate the physical, psychological, and behavioral characteristics of mobile phone users more comprehensively than if we had used the sole amount of time spent on mobile phone as a parameter. There are two possible interpretations of our results. On the one hand, users of various electronic equipment, such as phones and television, might be more exposed to SSF commercials [40,41]. College students who use mobile phones for longer may have more opportunities to buy SSF products, which might increase their SSF intake. On the other hand, as mentioned in the literature, participants of all ages choose SSF products as snacks rather than fruits, when viewing electronic equipment for an extended period of time [42]. Elucidating the mechanisms underlying the association between these behaviors, however, requires further investigation.

Moreover, as a potential factor, age also may influence SSF intake. Generally, with the increase in age, the level of education, experience and knowledge will increase accordingly, and people will become more mature and care about their health, thus decreasing their intake of SSF. Consistent with a previous study [43], with age, the intake of OSBB significantly decreased in both sexes, whereas the intake of sugar/chocolate significantly decreased in women only. This finding suggests that age is negatively associated with SSF intake and that SSF intake in women is more susceptible to age than in men. Presumably, with age, the education level and dietary knowledge of college students improve [44]. Women also are more concerned about their appearance than men [45], which causes them to reduce their SSF intake.

Additionally, we found that factors such as the NLAQ, total expenditure, food expenditure, and total physical activity were related to SSF intake. The application capacity of the NLAQ was negatively associated with sugar/chocolate intake in both sexes, which was in line with the results obtained by Buja et al. [46]. However, the comprehension capacity of the NLAQ was positively associated with sugar/chocolate intake in men; this may be explained by research conducted by Prada et al. [47], which indicated that participants with higher education understand the definition of free sugars better but do not necessarily apply this knowledge in practice. This behavior can be assimilated to that of smokers, who know that smoking is harmful to their health but continue to smoke because of the direct pressure exerted by their peers or the social environment [48]. This could explain why the male participants with a higher comprehension capacity had a high intake of sugar/chocolate. Therefore, this study demonstrated that the most important measure to reduce SSF intake in medical college students of different sexes is to improve their capacity to apply dietary health knowledge to their lives.

An unexpected finding was that the progressive increase in total and food expenditure per month further increased the intake of OSBB in both sexes. Based on the result of this study, the mean of total expenditure for this study population was 2160.39 CNY/month. The mean of total expenditure for Chinese college students was about 1700 CNY/month according to a research report published in 2020 [49], and the total expenditure of college students in Shanghai is higher than the national level in China. This study supported evidence from previous observations [27] that the participants consumed higher SSF when they had more pocket money in high-consumption areas. College students in high-consumption areas are more likely to buy SSF products when they have more money at their disposal. Furthermore, this study revealed that compared with low physical activity, high physical activity increased the intake of sugar/chocolate in male students. This result aligned with a previous study [28], which demonstrated that boys who spent less time performing moderate and high-intensity physical activity reported lower consumption of sugar-sweetened beverages and sugar than those who spent more time performing moderate and high-intensity physical activity. This result may be explained by the fact that vigorous-intensity exercise consumes a lot of energy [50]. In this context, sugar/chocolates are important sources of fast energy replenishment [51].

## 5. Strengths and Limitations

The present study has several strengths. First, it constituted the first description of the current pattern of SSF consumption and the association between the MPAI and SSF intake according to sex in medical college students from Shanghai, China. Therefore, it provides new evidence for further research on the MPAI and other potential factors that may influence SSF intake in medical college students, which requires more attention. Second, the sample size of this study was relatively large, the research subjects of medicine-related majors were relatively compliant, and the online questionnaire design included quality control to reduce potential bias. Third, the results of this study provide a scientific basis to reduce SSF consumption among medical college students of different sexes from Shanghai, China, including MPAI, NLAQ, and total and food expenditure.

Some limitations exist in our study. First, the full food-frequency questionnaire was not collected, which would not allow the calculation of the proportion of energy provided by SSF. This is an important issue to be addressed in future research. Second, because the study adopted cross-sectional design, causalities cannot be inferred. The real influence exerted by different factors on SSF intake in medical college students of different sexes is still uncertain. Third, because the study was conducted on a single campus of a university sample originating in Shanghai, and selected college students were all medicine-related majors, the research results cannot be extrapolated to all college students in China. Finally, some other factors that may influence SSF intake were not included in our study, such as the participants’ numbers of years attending college; future research needs to take these factors into consideration.

## 6. Conclusions

This study confirmed that SSF intake is widespread among medical college students from Shanghai, China, even if they have relatively high nutrition health literacy. From the public health perspective, SSF consumption should be decreased to prevent various related diseases in medical college students of Shanghai, China. This could be achieved by reducing the MPAI, controlling the total and food expenditure per month in high-consumption areas, and improving application capacity of the NLAQ in universities, family, and other environments. The research object of this study, however, came from only one campus of a university in Shanghai, China, and all students were enrolled in medicine-related majors. A considerable amount of work is needed to further determine the MPAI and other potential factors that may influence SSF intake by expanding the sample size of college students throughout China, and the causal association between them.

## Figures and Tables

**Table 1:** The demographic characteristics of total 1121 participants stratified by sex.

Characteristics	Male (*n* = 368)	Female (*n* = 753)	*p* Value
MPAI (score), Mean ± SD	44.94 ± 12.08	45.25 ± 11.87	0.682
Age (years), Mean ± SD	23.22 ± 3.32	23.45 ± 3.39	0.277
NLAQ (score)			
Acquisition capacity (score), Mean ± SD	12.44 ± 3.41	12.27 ± 3.37	0.431
Comprehension capacity (score), Mean ± SD	21.80 ± 4.12	21.99 ± 3.86	0.471
Application capacity (score), Mean ± SD	10.81 ± 2.64	10.93 ± 2.46	0.440
BMI (kg/m^2^)			
Normal, *n* (%)	240 (65.22)	541 (71.85)	<0.001
Underweight, *n* (%)	17 (4.62)	166 (22.05)	
Overweight and obese, *n* (%)	111 (30.16)	46 (6.11)	
Total physical activity (MET/min/w)			
<600 (MET/min/w), *n* (%)	43 (11.68)	188 (24.97)	<0.001
600–4000 (MET/min/w), *n* (%)	289 (78.53)	539 (71.58)	
≥4000 (MET/min/w), *n* (%)	36 (9.78)	26 (3.45)	
CB (mL/d)			
Low intake, *n* (%)	127 (34.51)	434 (57.64)	<0.001
High intake, *n* (%)	241 (65.49)	319 (42.36)	
OSBB (mL/d)			
Low intake, *n* (%)	205 (55.71)	399 (52.99)	0.391
High intake, *n* (%)	163 (44.29)	354 (47.01)	
Sugar/chocolate (g/day)			
Low intake, *n* (%)	246 (66.85)	438 (58.17)	0.005
High intake, *n* (%)	122 (33.15)	315 (41.83)	
Total expenditure (CNY/month)			
<2000 (CNY/month), *n* (%)	176 (47.83)	274 (36.39)	<0.001
≥2000 (CNY/month), *n* (%)	192 (52.17)	479 (63.61)	
Food expenditure (CNY/month)			
<1000 (CNY/month), *n* (%)	108 (29.35)	249 (33.07)	0.209
≥1000 (CNY/month), *n* (%)	260 (70.65)	504 (66.93)	

Abbreviations: MPAI, Mobile Phone Addiction Index; SD, standard deviation; NLAQ, Nutritional Literacy Assessment Questionnaire; BMI, Body Mass Index; CB, carbonated beverages; OSBB, other sugar-based beverages; MET, Metabolic Equivalent.

**Table 2:** Multivariate logistic model to explore MPAI and other potential factors that may influence carbonated beverages intake stratified by sex.

Characteristics	Male Adjusted OR (95%CI)	Female Adjusted OR (95%CI)
MPAI (score)	1.023 (1.004–1.042) *	1.026 (1.013–1.039) *
Age (years)	0.979 (0.915–1.048)	0.958 (0.916–1.003)
NLAQ (score)		
Acquisition capacity	0.954 (0.893–1.020)	1.045 (0.999–1.094)
Comprehension capacity	1.015 (0.952–1.081)	1.039 (0.993–1.088)
Application capacity	1.007 (0.911–1.112)	1.012 (0.942–1.089)
BMI (kg/m^2^)		
Normal	1 (Reference)	1 (Reference)
Underweight	0.850 (0.292–2.474)	1.144 (0.798–1.641)
Overweight and obese	1.149 (0.702–1.880)	1.495 (0.807–2.773)
Total physical activity (MET/min/w)		
<600 MET/min/w	1 (Reference)	1 (Reference)
600–4000 MET/min/w	0.880 (0.427–1.815)	0.956 (0.679–1.348)
≥4000 MET/min/w	0.771 (0.291–2.046)	0.687 (0.285–1.652)
Total expenditure (CNY/month)		
<2000 CNY/month	1 (Reference)	1 (Reference)
≥2000 CNY/month	0.868 (0.516–1.463)	1.308 (0.912–1.875)
Food expenditure (CNY/month)		
<1000 CNY/month	1 (Reference)	1 (Reference)
≥1000 CNY/month	1.881 (1.078–3.281) *	1.092 (0.756–1.577)

Abbreviations: MPAI, Mobile Phone Addiction Index; OR, odds ratio; CI, confidence interval; NLAQ, Nutritional Literacy Assessment Questionnaire; BMI, Body Mass Index; MET, Metabolic Equivalent. * *p* < 0.05.

**Table 3:** Multivariate logistic model to explore MPAI and other potential factors that may influence other sugar-based beverages intake stratified by sex.

Characteristics	Male Adjusted OR (95%CI)	Female Adjusted OR (95%CI)
MPAI (score)	1.019 (1.001–1.038) *	1.020 (1.007–1.033) *
Age (years)	0.929 (0.865–0.997) *	0.948 (0.904–0.993) *
NLAQ (score)		
Acquisition capacity	1.023 (0.957–1.093)	1.034 (0.987–1.082)
Comprehension capacity	1.054 (0.989–1.123)	1.010 (0.965–1.057)
Application capacity	0.994 (0.900–1.098)	0.978 (0.910–1.052)
BMI (kg/m^2^)		
Normal	1 (Reference)	1 (Reference)
Underweight	0.631 (0.220–1.815)	1.081 (0.750–1.557)
Overweight and obese	0.830 (0.511–1.349)	1.687 (0.893–3.187)
Total physical activity (MET/min/w)		
<600 MET/min/w	1 (Reference)	1 (Reference)
600–4000 MET/min/w	0.716 (0.353–1.454)	0.763 (0.540–1.080)
≥4000 MET/min/w	0.859 (0.331–2.226)	0.448 (0.185–1.088)
Total expenditure (CNY/month)		
<2000 CNY/month	1 (Reference)	1 (Reference)
≥2000 CNY/month	1.750 (1.060–2.890) *	1.991 (1.387–2.857) *
Food expenditure (CNY/month)		
<1000 CNY/month	1 (Reference)	1 (Reference)
≥1000 CNY/month	2.441 (1.378–4.324) *	1.598 (1.106–2.309) *

Abbreviations: MPAI, Mobile Phone Addiction Index; OR, odds ratio; CI, confidence interval; NLAQ, Nutritional Literacy Assessment Questionnaire; BMI, Body Mass Index; MET, Metabolic Equivalent. * *p* < 0.05.

**Table 4:** Multivariate logistic model to explore MPAI and other potential factors that may influence sugar/chocolate intake stratified by sex.

Characteristics	Male Adjusted OR (95%CI)	Female Adjusted OR (95%CI)
MPAI (score)	1.005 (0.986–1.025)	1.019 (1.006–1.032) *
Age (years)	0.942 (0.872–1.017)	0.948 (0.906–0.993) *
NLAQ (score)		
Acquisition capacity	0.996 (0.931–1.067)	1.012 (0.967–1.059)
Comprehension capacity	1.082 (1.013–1.155) *	1.035 (0.989–1.083)
Application capacity	0.901 (0.813–0.998) *	0.909 (0.846–0.977) *
BMI (kg/m^2^)		
Normal	1 (Reference)	1 (Reference)
Underweight	1.006 (0.346–2.927)	1.377 (0.963–1.967)
Overweight and obese	0.716 (0.430–1.192)	0.799 (0.421–1.516)
Total physical activity (MET/min/w)		
<600 MET/min/w	1 (Reference)	1 (Reference)
600–4000 MET/min/w	1.595 (0.728–3.493)	1.026 (0.728–1.448)
≥4000 MET/min/w	2.860 (1.047–7.812) *	0.798 (0.336–1.895)
Total expenditure (CNY/month)		
<2000 CNY/month	1 (Reference)	1 (Reference)
≥2000 CNY/month	1.042 (0.622–1.748)	1.206 (0.842–1.727)
Food expenditure (CNY/month)		
<1000 CNY/month	1 (Reference)	1 (Reference)
≥1000 CNY/month	1.648 (0.915–2.967)	0.846 (0.587–1.219)

Abbreviations: MPAI, Mobile Phone Addiction Index; OR, odds ratio; CI, confidence interval; NLAQ, Nutritional Literacy Assessment Questionnaire; BMI, Body Mass Index; MET, Metabolic Equivalent. * *p* < 0.05.

## Data Availability

The data presented in this study are available on request from the corresponding author. The data are not publicly available due to privacy of study participants.

## Supplementary Materials

The following are available online at https://www.mdpi.com/article/10.3390/nu13072256/s1, Table S1: Factor analysis of Mobile Phone Addiction Index, Table S2: Factor analysis for Nutrition Literacy Assessment Questionnaire.
