# Supplementary material for: Association between Mobile Phone Addiction Index and Sugar-Sweetened Food Intake in Medical College Students Stratified by Sex from Shanghai, China
Source: Nutrients. 2021 Jun 30;13(7):2256. doi: 10.3390/nu13072256 (PMC8308374; doi:10.3390/nu13072256)
Supplement: Supplementary file 1 [file nutrients-13-02256-s001.zip › nutrients-1254328-supplementary.pdf]

Table S1 Factor analysis of Mobile Phone Addiction Index

	Factors					
	**1**	**2**	**3**	**4**	**Mean**	**SD**
**Inability to control craving**						
Your friends and family complained about your use of the mobile phone	0.772				2.06	1.23
You have been told that you spend too much time on your mobile phone	0.746				1.97	1.18
You have tried to hide from others how much time you spend on your mobile phone (7)*	0.66				1.89	1.02
You have received mobile phone bills you could not afford to pay	0.61				2.17	1.39
You find yourself engaged on the mobile phone for longer period of time than intended (5)*	0.591				2.49	1.19
You have attempted to spend less time on your mobile phone but are unable to (3)*	0.589				1.98	1.09
You can never spend enough time on your mobile phone (2)*	0.583				2.09	1.07
**Feeling anxious and lost**						
When out of range for some time, you become preoccupied with the thought of missing a call (1)*		0.72			2.62	1.27
You find it difficult to witch off your mobile phone		0.713			2.57	1.42
You feel anxious if you have not checked for messages or switched on your mobile phone for some time (4)*		0.691			2.5	1.27
You feel lost without your mobile phone		0.671			2.77	1.36
If you don't have a mobile phone, your friends would find it hard to get in touch with you		0.663			3.62	1.29
**Withdrawal/escape**						
You have used your mobile phone to talk to others when you were feeling isolated			0.847		2.64	1.27
You have used your mobile phone to talk to others when you were feeling lonely			0.786		3.08	1.28
You have used your mobile phone to make yourself feel better when you were feeling down (8)*			0.62		3.21	1.29
**Productivity loss**						
You find yourself occupied on your mobile phone when you should be doing other things, and it causes a problem (6)*				0.818	1.86	0.97
Your productivity has decreased as a direct result of the time you spend on the mobile phone				0.771	2.06	1.04
Eigenvalue	6.63	1.47	1.15	1.03		
Variance explained	39.01	8.63	6.76	6.03		
Cronbach's alpha	0.84	0.8	0.81	0.62		

Notes: Scale used: 1=not at all; 2=rarely; 3=occasionally; 4=often; and 5=always. N=402. *Items resemble or are equivalent to the 8-item Young's Internet addiction diagnostic scale.

Table S2 Factor analysis for Nutrition Literacy Assessment Questionnaire

	Factor loadings		
	1	2	3
I am vulnerable to nutritional dietary advice on new media (wechat, Weibo)	**0.828**	-0.049	0.031
If I discuss food with others, I often refer to the information in the media	**0.779**	-0.086	0.007
I mainly learn nutrition and diet knowledge from new media (Wechat and weibo)	**0.713**	-0.079	0.028
I believe in the various dietary suggestions I read in the media	**0.697**	0.257	-0.154
I think the dietary guidelines are easy to understand	0.024	**0.800**	0.070
I understand the concept of “balanced diet”	0.128	**0.739**	-0.014
I can understand the information on food labels (nutrients and energy, etc.)	0.074	**0.730**	0.115
When I read about nutrition and diet, I don’t need someone to help me understand it	-0.190	**0.705**	-0.119
I found the nutritionist's statement easy to understand	-0.169	**0.680**	-0.085
I understand the core items and standards in the dietary guidelines	0.106	**0.633**	0.090
I refer to the label information on the food package (nutrients, energy, etc.) when choosing food	0.001	0.055	**0.811**
I can choose food according to my own nutritional status (such as fat free, less sugar, etc.)	-0.046	0.067	**0.795**
I'm willing to spend extra time or money on healthy meals	-0.023	-0.067	**0.763**
Eigen value	2.604	3.495	1.407
Variance explained（%）	20.028	26.883	10.822
Cumulative sums of squares（%）	46.911	26.883	57.733
